# Supplementary figures and images for: The Endocrine-Disrupting Chemical Benzophenone-3 in Concentrations Ranging from 0.001 to 10 µM Does Not Affect the Human Decidualization Process in an In Vitro Setting
Source: Int J Mol Sci. 2025 Sep 24;26(19):9314. doi: 10.3390/ijms26199314 (PMC12525503; doi:10.3390/ijms26199314)

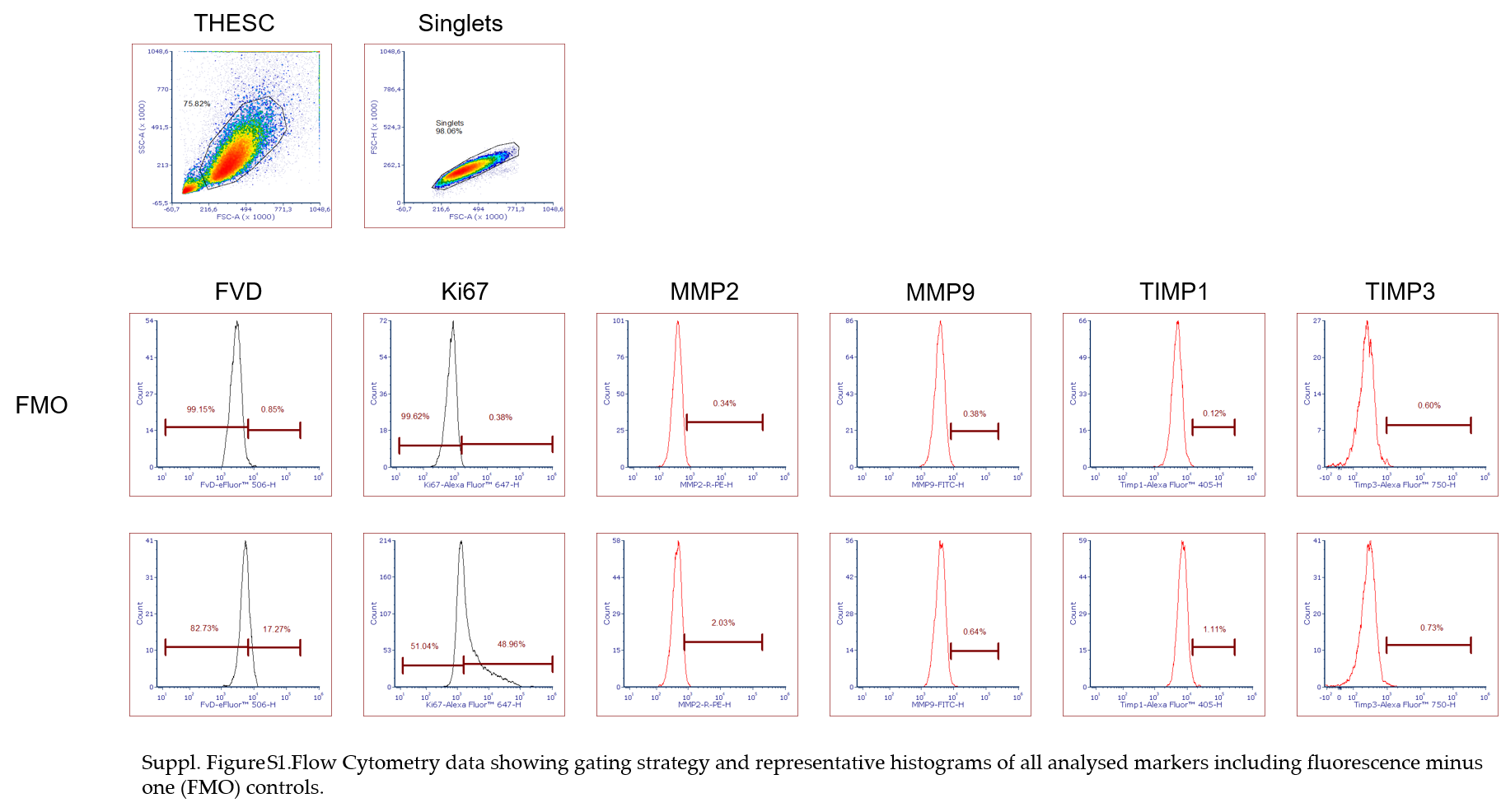

Supplement: Supplementary file 1 [file ijms-26-09314-s001.zip › ijms-3781737-supplementary.tif]
